# Effects of trunk training using motor imagery on trunk control ability and balance function in patients with stroke

**DOI:** 10.1186/s13102-023-00753-w

**Published:** 2023-10-26

**Authors:** Yan-fang Sui, Zhen-hua Cui, Zhen-hua Song, Qian-qian Fan, Xia-fei Lin, Binbin Li, Liang-qian Tong

**Affiliations:** 1 Department of Rehabilitation Medicine, Affiliated Haikou Hospital of Xiangya Medical College, Central South University, Haikou, 570208 China; 2 Department of nuclear medicine, Affiliated Haikou Hospital of Xiangya Medical College, Central South University, No.43 of Renmin Road, Haikou District, Haikou, 570208 China

**Keywords:** Balance function, Motor imagery, Stroke; surface electromyography (sEMG), Trunk training

## Abstract

**Objective:**

To explore the effects of trunk training using motor imagery on trunk control and balance function in patients with stroke.

**Methods:**

One hundred eligible stroke patients were randomly divided into a control group and trial group. The control group was given routine rehabilitation therapy, while the trial group was given routine rehabilitation therapy and trunk training using motor imagery.

**Results:**

Prior to treatment, there was no significant difference between the two groups (*P > 0.05*) in Sheikh’s trunk control ability, Berg rating scale (BBS), Fugl-Meyer assessment (FMA), movement length, movement area, average front-rear movement speed, average left-right movement speed, and surface electromyography (sEMG) signal of the bilateral erector spinae and rectus abdominis. After treatment, Sheikh’s trunk control ability, FMA, and BBS in the two groups were significantly higher than those before treatment (*P < 0.05*). The movement length, movement area, the average front-rear movement speed, and the average left-right movement speed in the two groups decreased significantly (*P < 0.05*). The differences of these indicators between the two groups were statistically significant (*P < 0.05*). After treatment, the rectus abdominis and erector spinae on the affected side of the two groups improved when compared with those before treatment (*P < 0.05)*. The rectus abdominis and erector spinae on the healthy side of the trial group descended after treatment (*P < 0.05*), while little changes were observed on the healthy side of the control group after treatment (*P > 0.05)*. The rectus abdominis and erector spinae on the affected side of the trial group improved when compared with those in the control group (*P < 0.05*). There was no significant difference between the two groups in the decline of abdominalis rectus and erector spinal muscle on the healthy side.

**Conclusion:**

Trunk training using motor imagery can significantly improve the trunk control ability and balance function of stroke patients and is conducive to promoting the recovery of motor function.

## Introduction

Stroke has a high disability rate and can lead to motor, sensory, cognitive, and psychological disorders, of which motor dysfunction is the main cause of disability in stroke patients, seriously affecting the physical activities and quality of life of patients [1]. Therefore, improving the motor function of patients with stroke is one of the main goals of rehabilitation. Motor imagery therapy is a treatment method where the patient mentally rehearses movement (motor task) repeatedly without any accompanied physical movement, to improve motor function [2–4]. In recent years, motor imagery therapy has attracted extensive clinical attention as an emerging treatment [5]. Previous studies mainly explored the effects of motor imagery therapy on post-stroke limb function recovery [6–10]. However, stroke is often accompanied by trunk dysfunction. The trunk is at the center of the human body and is the basis of physical activity and plays a role in adjusting the center of gravity. Patients with stroke show decreased and delayed muscle activity in the paralyzed trunk; however, the stability of the trunk in stroke patients depends on muscle strength, neural control, and appropriate proprioceptive sensibility [11]. Patients with stroke cannot maintain a balanced and stable posture, and losing balance is an important factor leading to falls, thus slowing the rate of recovery. Therefore, trunk functional recovery is a precursor for patients to recover various functions. However, there are few reports on the effect of motor imagery therapy on trunk control after stroke in China or abroad. In this study, we observed the effects of trunk training using motor imagery on trunk control ability, balance function, and motor function in patients with stroke.

## Data and methods

### General information

Post-stroke patients with motor dysfunction hospitalized in the Department of Rehabilitation Medicine of our hospital from January 1,2020 to January 1,2022, aged 50 to 70 years old, were selected.

Inclusion criteria: [12] ① The patient met the diagnostic criteria for stroke established at the Fourth National Academic Conference on Cerebrovascular Diseases in 1995, [13, 14] and stroke was diagnosed as the primary disease by CT or MRI. ② The time between disease onset and enrollment ranged between two weeks and three months. ③ The patient’s vital signs were stable, and the patient was conscious, could understand the instructions, and cooperate with rehabilitation training. ④ The patient’s kinesthetic and visual imagery questionnaire (KVIQ) score was ≥ 25 points. ⑤ The patient signed the required informed consent form. ⑥ Aged between 50 and 70 years.

Exclusion criteria: [14] ① The patient suffered from severe cardiopulmonary, hepatic, or renal insufficiency, malignant tumor, etc. ② The patient suffered from impaired consciousness, aphasia, mental disorder, or major cognitive dysfunction. ③ The patient has other craniocerebral diseases or traumatic sequelae in the past. ④ The patient has previous severe osteoarticular diseases causing abnormal trunk function.

Finally, a total of 100 stroke patients with motor dysfunction were included, and they were divided into a control group and a trial group according to the random number table, with 50 cases in each group. There was no significant difference (*P > 0.05*) in general data such as sex, age, course of disease, and KVIQ between the two groups, and they were comparable. See Table 1 for details. This study was approved by the local ethics committee (Approval number: 2018-ethical review-189) and performed in accordance with the Declaration of Helsinki. All participants provided written informed consent.

**Table 1:** Comparison of general data of patients such as sex, age, course of disease, and lesion site between the two groups

Group	Number of cases	Sex (cases)		Age (years old, —x ±s)	Hemiplegic side		Course of a disease (days $\bar x \pm s$)	KVIQ (points)
		Male and female			Right Left			
Control group	50	31	19	58.90 ± 4.78	34	16	55.37 ± 22.24	93.12 ± 7.18
Trial group	50	32	18	59.50 ± 4.80	33	17	56.13 ± 23.54	94.23 ± 6.98

Note: There were no significant differences in sex, age, course of disease, or KVIQ between the two groups (*P > 0.05*)

### Treatment methods

The patients in the control group underwent routine rehabilitation therapy and maintained a supine position for an equivalent duration in the same environment as the combined trunk motor imagery therapy. Meanwhile, the trial group received both routine rehabilitation therapy and combined trunk motor imagery therapy.

#### Routine rehabilitation therapy

The training included good limb positioning, neuromuscular promotion techniques, such as proprioceptive neuromuscular facilitation (PNF) technique, Rood’s approach, motor relearning, occupational therapy, daily living ability training, and traditional therapy. The participants received routine rehabilitation therapy for a duration of five hours per day, five times a week, over a period of four weeks.

#### Motor imagery therapy

The training of motor imagery therapy consisted of six steps: [4, 14] ① Task illustration: The therapist first demonstrated and explained the contents of the imagery training, asked the patients to carefully observe and identify the part of the limb that was “active,“ what kind of movement had to be done, and master the normal movement and feeling. ② Preview: the patients were asked to imagine the relevant movements again. ③ Motor imagery: the patients listened to the motor imagery instruction tape and practiced the imagery. ④ Rehabilitation training: the patients repeatedly practiced the movements of imagery training. ⑤ Problem solving: the patients learned relevant skills through repeated practice. ⑥ Practical application: the patients turned relevant skills into practical skills. Prior to motor imagery, a video of a normal person’s trunk movement was shown, including steady trunk movement with a Bobath ball, and balance movements while sitting, standing, and reaching out to move a water cup. The 10-minute video along with audio was shown to the patients via a computer in a quiet treatment room. During each training session, the patients were instructed to close their eyes and sit on a comfortable chair with their body relaxed. The patients then imagined the movement of their body based on the specific motor imagery instructions in the video. During treatment, the therapist interrupted the patients intermittently to ask questions, to see if they can concentrate on the imagery of the physical movement. At the end of session, the patients were asked to refocus their attention on their surroundings, following which, they were sent back to their room and asked to feel their physical being. Then, the patients were asked to pay attention to the ambient sounds. Finally, the narrator counted down from 10 to 1, and the patients were asked to open their eyes when the countdown reached 1. A motor imagery video was provided only during the first treatment, after which the patients underwent motor imagery training according to the motor imagery guidelines. The motor imagery therapy sessions were conducted for 30 min each time, with a frequency of five times per week, for a total of four weeks.

### Observation indicators and evaluation methods

The patient’s trunk control evaluation was conducted before treatment and four weeks after treatment using Sheikh trunk control evaluation. The simple Fugl-Meyer assessment (FMA), Berg rating scale (BBS), and balance feedback trainer were used to evaluate the motor and balance functions of the patients. In addition, before and after treatment, the sEMG signals of the bilateral erector spinae and rectus abdominis in the maximum range of flexion and extension at a uniform speed under the sitting position were measured by sEMG signals. All evaluations were performed by the same evaluator in a blinded manner.

#### Sheikh trunk control evaluation

Sheikh is a scale for evaluating trunk control ability. It includes four movements of turning from the supine position to hemiplegic side, turning to the healthy side, sitting up from the supine position, and maintaining balance in a sitting position on the bed. The scoring method is: 0 points for non-completion, 12 points for completion but requiring some help (grabbing or leaning on an object), and 25 points for normal completion. A higher total score indicates better trunk control.

#### BBS rating

The balance function is divided into 14 items from easy to difficult, and each item is scored based on a five-point scale—0, 1, 2, 3, and 4. The highest score is 4 points, and the lowest is 0 points. The highest integral score is 56 points, and the lowest is 0 points. The higher the score, the better the balance function.

#### Motor function evaluation

FMA is used to evaluate the motor function of patients. The highest score is 100. The higher the score, the better the motor function of patients will be.

#### Evaluation of balance feedback training apparatus

The ProKin 254P (PK-254P) balance feedback training apparatus manufactured by TecnoBody Ltd., Italy, was used to test the posture stability of the patients. Stability testing was performed in open-eye standing position using the static mode of the PK-254P balancer. Standard standing posture includes: ① Standing bilaterally symmetrically with A1A5 as the center axis. ② The patients holds out their head high and look straight ahead. ③ Both upper limbs are naturally placed on both sides of the body. ④ The medial edges of both feet are 10 cm apart, and the highest point of the bilateral arches is located on axis A3A5. Observation parameters are as follows: movement length, movement area, average front-rear movement speed, and average left-right movement speed.

#### sEMG signal acquisition

While the patients are sitting on a square stool, their trunk is subjected to anterior flexion and posterior extension in the maximum range with uniform speed. The Shanghai NCC 8-channel sEMG signal acquisition system was used for acquiring the bilateral erector spinae and rectus abdominis myoelectric signals. The electrode pads were pasted on the 3 cm lateral opening at the left and right side of L3 spinous process (erector spinae) and the 3 cm lateral opening at the left and right side 3 cm above the navel (rectus abdominis). The conductive diameter of the electrodes was l cm, and the electrode pitch was 2 cm. Dandruff and oil were removed with a fine gauze and alcohol before testing. The root mean square (RMS) of myoelectric signals was then analyzed. The trial was repeated three times with an interval of 30 s to obtain the average value. The RMS of the bilateral rectus abdominis and erector spinae of the two groups were evaluated before treatment and four weeks after treatment.

### Statistical analysis

SPSS software version 16.0 was used to analyze the data. The measurement data are expressed as ($\bar x \pm s$). Parametric statistics were applied when the collected data satisfied the assumptions of homogeneity of variance and normal distribution. When these assumptions were not met, non-parametric statistics were used. The paired sample T test was used for pre-treatment and post-treatment comparison within the same group, while the independent sample T test was used for inter-group comparison, and *P* < 0.05 indicated that the difference was statistically significant.

## Results

There were no significant differences in each parameter before treatment between the groups. Before treatment, there was no significant difference in Sheikh, BBS, and FMA scores between the two groups (*P > 0.05*). At the follow-up 4 weeks after the end of the treatment, the Sheikh, BBS, and FMA scores of the two groups were significantly higher than those before treatment, and the differences were statistically significant (*P < 0.05*). The difference between groups was statistically significant (*P < 0.05)*. See Table 2 for details.

**Table 2:** Comparison of Sheikh, FMA, and BBS scores between the two groups before treatment and four weeks after treatment (points, $\bar x \pm s$)

	Sheikh score	BBS score	FMA score
Control group			
Before treatment	35.67 ± 5.63	11.72 ± 2.27	29.44 ± 4.26
After treatment	41.22 ± 5.93^a^	18.94 ± 1.73^a^	36.33 ± 3.60^a^
Trial group			
Before treatment	37.56 ± 4.83	11.67 ± 2.40	29.94 ± 5.05
After treatment	60.28 ± 5.64^ab^	21.89 ± 3.18^ab^	40.05 ± 3.78^ab^

Note: After four weeks of treatment, compared with the status before treatment within the same group, ^a^P < 0.05 was observed. After four weeks of treatment, the ^b^P < 0.05 was observed in the inter-group comparison

Before treatment, the differences in movement length, movement area, average front-rear motor speed, and average left-right motor speed in the sitting position between the two groups were not statistically significant (*P > 0.05*). Four weeks after treatment, the motor length, motor area, average front-rear motor speed, and average left-right motor speed in the sitting position of the patients between the two groups were significantly lower than before, and the differences were statistically significant (*P < 0.05*). After four weeks of treatment, there were significant differences in all indexes between the trial group and the control group (*P < 0.05*). See Table 3 for details.

**Table 3:** Comparison of movement length, movement area, and average movement speed between the two groups before and after treatment

		Average left and right exercise speed (mm/s)	Average front-rear exercise speed (mm/s)	Motion length (mm)	Moving area (mm^2^)
Control group					
Before treatment	50	16.24 ± 4.18	15.93 ± 4.12	571.12 ± 165.35	754.06 ± 259.16
After treatment	50	13.78 ± 3.03^a^	12.08 ± 3.04^a^	463.38 ± 122.54^a^	629.34 ± 206.02^a^
Trial group					
Before treatment	50	15.75 ± 4.11	15.04 ± 4.25	571.56 ± 179.43	747.09 ± 269.89
After treatment	50	9.15 ± 3.01^ab^	9.86 ± 3.15^ab^	438.86 ± 118.28^ab^	516.42 ± 178.56^ab^

Note: Comparison within the same group before treatment, ^a^P < 0.05 was observed, and compared with the control group, ^b^P < 0.05 was observed

Before treatment, the bilateral rectus abdominis and erector spinae were compared between the two groups, (*P > 0.05)*. After treatment, the rectus abdominis and erector spinae on the affected side of the two groups improved when compared with those before treatment (*P < 0.05)*. The rectus abdominis and erector spinae on the healthy side of the trial group descended after treatment (*P < 0.05*), while little changes were observed on the healthy side of the control group after treatment (*P > 0.05)*. The rectus abdominis and erector spinae on the affected side of the trial group improved when compared with those in the control group (*P < 0.05*). There was no significant difference between the two groups in the decline of abdominalis rectus and erector spinal muscle on the healthy side. See Table 4 for details.

**Table 4:** Comparison of RMS values of the bilateral rectus abdominis and erector spinae (µV, $\bar x \pm s$)before and after four weeks of treatment

	Affected side of control group		Healthy side of control group		Affected side of trial group		Healthy side of trial group	
	Rectus abdominis	Erector spinae	Rectus abdominis	Erector spinae	Rectus abdominis	Erector spinae	Rectus abdominis	Erector spinae
Before treatment	12.13 ± 5.58	13.56 ± 5.68	22.52 ± 7.56	24.93 ± 6.68	12.37 ± 6.63	13.25 ± 5.34	23.73 ± 8.01	25.82 ± 6.88
After treatment	16.46 ± 5.67	16.82 ± 5.26	21.73 ± 6.48	23.15 ± 6.25	20.76 ± 5.23	21.82 ± 5.34	21.24 ± 7.34	22.85 ± 6.23

Note: Before treatment, the comparison of the bilateral rectus abdominis and erector spinae between the two groups showed *P* > 0.05. After four weeks of treatment, the bilateral rectus abdominis and erector spinae of the trial group were compared with those before treatment (*P < 0.05*). The rectus abdominis and erector spinae on the affected side of the control group were compared with those before treatment (*P < 0.05*), while the rectus abdominis and erector spinae on the healthy side of the control group were compared with those before treatment (*P > 0.05*). After four weeks of treatment, the rectus abdominis and erector spinae on the affected side of the trial group were compared with those of the control group (*P* < 0.05). The rectus abdominis and erector spinae on the healthy side of the trial group were compared with those of the control group (*P > 0.05*)

## Discussion

Motor imagery therapy refers to repeated mental imagery of a certain action or motor scenario under the guidance of implicature-based instructions, but without any physical output, and the activation of a specific area in the brain based on the motor memory, to achieve the purpose of improving the motor function [15–18]. It is theoretically modelled on the psychoneuromuscular theory (PM theory), which suggests that real motion and motor imagery have similar motor neuron pathways. By training the motor neurons and the “motor pattern” stored in the motor cortex, the motor imagery can achieve the same effect as real motion. Motor imagery and real motion have similar neural mechanism and can activate brain regions in a similar manner as real motion [19, 20]. Motor imagery refers to the absence of physical movement, while the memory of the physical action is mentally rehearsed using a dynamic process. Repeated motor imagery training can promote functional recovery through neural plasticity [21]. Motor imagery has been used for neurorehabilitation in patients, such as those after cervical spinal cord injury or stroke, to promote brain plasticity [22–24]. The application of motor imagery has also been explored in athletes [25].

The results of this study showed that after four weeks of treatment, the Sheikh scores of trunk control ability in the two groups were both improved compared with before, but the improvement in the trial group was more significant, and the difference was statistically significant (*P < 0.05*), which indicated that motor imagery training could significantly improve the trunk control ability of the patients. In addition, four weeks after treatment, the FMA and BBS scores of the two groups were also higher than before treatment, the improvement in the trial group was more significant, and the difference was statistically significant (*P < 0.05*). After four weeks of treatment, the movement length, movement area, average front-rear movement speed, and average left-right movement speed in the two groups decreased, and the difference was statistically significant (P *< 0.05*). Compared with the control group, all indexes of the trial group were significantly increased (P *< 0.05*), suggesting that trunk training using motor imagery can also improve the patient’s sitting balance and motor function. The results of this study are consistent with those of Oh et al. [26]. According to the research results, motor imagery training can improve the trunk control ability of patients with stroke. In the past, most studies on motor imagery focused on the motor imagery of limbs. The studies confirmed that motor imagery can improve the motor function of limbs in patients with stroke, without the need to focus on the core muscle groups of the trunk. Based on previous studies, we performed motor imagery training for the trunk and observed the effect on the control ability of the trunk and on the improvement of the balance and motor function.

The trunk is at the core of the human body, and is the basis for the body to maintain support, move and adjust the center of gravity—only by adjusting the center of gravity is it possible to maintain balance [27]. The trunk motor muscle is dominated by bilateral neurons, so there is no obvious hemiplegia after stroke. However, the research by Bohannon et al. [28] showed that the muscle strength on the hemiplegic side decreased by 30% compared with that on non-hemiplegic side, and it decreased in many directions, in particular the body flexion decreased significantly. Moreover, previous studies suggest that both the left and right cerebral lesions could decrease the control ability of trunk muscles, and there is no significant difference between the two [29]. Therefore, trunk control training should be strengthened after stroke. The results of this study revealed that after stroke, there was a significant imbalance in the sEMG signals of the bilateral erector spinae and rectus abdominis, and the sEMG signals of the non-hemiplegic side were significantly superior to that of the hemiplegic side, indicating that muscle strength of the erector spinae and rectus abdominis on the non-hemiplegic side was significantly superior to that on the hemiplegic side after stroke. This result is consistent with a previous study [19]. After trunk training using motor imagery, the sEMG signals of the hemiplegic side in the trial group were significantly higher than those before treatment *(P* < *0.05)*. In the control group, the improvement of sEMG signals on the hemiplegic side was not significant as compared with that before treatment (*P > 0.05*). The results show that trunk training using motor imagery could significantly improve trunk muscle strength of patients with stroke and significantly improve the imbalance of muscle strength on both sides. Previous studies suggest that core stability training using motor imagery can improve the balance function and walking ability of patients with post-stroke hemiplegia [19]. It has also been proven that motor imagery therapy can improve the balance function and daily life activities of patients in the initial stage of stroke [30–32].

The present study is limited by small sample size, single-center design, and short follow-up. Further investigations are needed to verify the current findings.

## Conclusion

Trunk training using motor imagery is a new rehabilitation therapy, and it needs to be further standardized and improved through clinical trials, and its mechanism needs to be further explored in future clinical trials.

## Data Availability

The datasets used and/or analysed during the current study available from the corresponding author on reasonable request.

## Abbreviations

BBS: Berg rating scale
FMA: Fugl-Meyer assessment
sEMG: Surface electromyography
KVIQ: Kinesthetic and visual imagery questionnaire
PNF: Proprioceptive neuromuscular facilitation
RMS: Root mean square
