# Correction: Effects of trunk training using motor imagery on trunk control ability and balance function in patients with stroke

**DOI:** 10.1186/s13102-025-01269-1

**Published:** 2025-07-24

**Authors:** Yan-fang Sui, Zhen-hua Cui, Zhen-hua Song, Qian-qian Fan, Xia-fei Lin, Binbin Li, Liang-qian Tong

**Affiliations:** 1 Department of Rehabilitation Medicine, Affiliated Haikou Hospital of Xiangya Medical College, Central South University, Haikou, 570208 China; 2 Department of nuclear medicine, Affiliated Haikou Hospital of Xiangya Medical College, Central South University, No.43 of Renmin Road, Haikou District, Haikou, 570208 China

**Correction:** ***BMC Sports Sci Med Rehabil*** **15, 142 (2023)**

10.1186/s13102-023-00753-w

Following publication of the original article [1], the corresponding author’s name has been corrected from Lang-qian Tong to Liang-qian Tong.

The original article [1] has been updated.
